# Sustainable Development Goal Integration, Interdependence, and Implementation: the Environment–Economic–Health Nexus and Universal Health Coverage

**DOI:** 10.1002/gch2.201900021

**Published:** 2019-04-29

**Authors:** Marlon E. Cerf

**Affiliations:** ^1^ Grants, Innovation and Product Development South African Medical Research Council PO Box 19070 Tygerberg Cape Town 7505 South Africa; ^2^ Biomedical Research and Innovation Platform South African Medical Research Council PO Box 19070 Tygerberg Cape Town 7505 South Africa

**Keywords:** global health strategy, health services, low‐ and middle‐income countries

## Abstract

The sustainable development goals (SDGs) are interdependent and integrated. The SDGs aim to impact all levels of society, reach across all sectors, embrace equity, inclusion, and universality, and operate in an ecosystem. SDG 3 strives to improve health outcomes by reducing mortality, ending epidemics, preventing diseases, and implementing universal health coverage (UHC) for accessible, affordable, quality, and equitable healthcare. The available infrastructure (environment) should be supportive and sustained by a stable, growing economy (economy) to ensure the timely and reliable delivery of quality healthcare (health). A general framework is presented for the implementation of the SDGs with a focus on SDG 3 and one of its targets, UHC. The robustly aligned environment–economic–health nexus is a key determinant for the successful implementation of UHC (and the SDGs). SDG implementation presents multiple convoluted challenges at national, regional, continental, and global levels but will ultimately yield rewards. To advance UHC, a global effort is required where timely information is shared and nations partner to achieve this important global health target. Especially in low‐ and middle‐income countries (LMICs), the learning and sharing of experiences and knowledge are essential throughout the course of implementation. This will ensure that no nation is left behind.

## 1 The Sustainable Development Goals

### 1.1 Sustainable Development Goals and Targets

The sustainable development goals (SDGs) present the opportunity to strengthen health governance, assuming that purposeful action informs governance in other policy areas to promote and protect health.^1^ Sustainable energy, sustainable consumption and production, food security, income inequality, migration, and trade and intellectual property all present policies of particular relevance^1^ for reinforcing health governance. The SDGs are integrated and even with a focus on health, the contribution of the other SDGs, some directly and others more indirectly, requires consideration.

The 13 targets and 26 indicators of SDG 3 focus on promoting healthy lifestyles and well‐being for all.^1^ Briefly the health SDGs strive to reduce (morbidity and) mortality of mothers (3.1) and children (3.2), end communicable disease epidemics (3.3), reduce deaths from noncommunicable diseases (NCDs) (3.4), enhance the prevention and treatment of substance abuse (3.5), reduce traffic injury mortality (3.6), ensure universal access to reproductive healthcare services (3.7) and health coverage (universal health coverage (UHC)) (3.8), and to reduce the morbidity and mortality from harmful substances and pollutants (3.9).^2^ Further, there are the additional goals of strengthening the implementation of the World Health Organization (WHO) Framework Convention on Tobacco (3a), support R&D for vaccines and medicine for communicable diseases and NCDs (3b), increase health resourcing (3c), and strengthen the capacity for early detection of health risks (3d).^2^

### 1.2 Sustainable Development Goal Integration, Interdependence, and the Environment–Economic–Health Nexus

SDG integration and interdependence are critical for advancement and successful implementation of individual and collective SDGs. Integration refers to each SDG viewed in the context of the other SDGs, i.e., not in isolation. Advancing an individual SDG, e.g. SDG 3, therefore requires consideration of the other SDGs which operate in the SDG ecosystem. An exclusive focus on a specific SDG without considering the other SDGs will constrain progress. Each individual SDG is an input into the collective SDG ecosystem for greater synergism to facilitate progress. Interdependence refers to an individual SDG's reliance on directly and indirectly related SDGs to meet targets. Progress in one SDG ripples to progress in related SDGs that can be leveraged throughout the SDG ecosystem for all‐encompassing advancement across all SDGs. For advancing health (SDG 3), the supporting infrastructure (environment) and adequate economic growth are necessary.

To illustrate the integration and interdependence of the SDGs, a brief overview of some environmental, economic, and health factors is presented. Environmental disasters and events disrupt the health and economic landscapes.^3^ For example, a severe drought (environment) may latently prompt famine and exacerbate poverty ((socio)economic) that is attributed to rising food inflation (economic) while also increasing the incidence of infections (health) due to water scarcity and impurity. Further, cyclones (environment) and flash flooding (environment) drive the prioritization and reallocation of public funds (economic) for aiding displaced individuals and for restoring infrastructure possibly at the expense of healthcare (health) and welfare.^3^

Expanding on this environment–economic–health nexus, the available infrastructure (environment) should be enabling and needs to be sustained by a stable growing economy (economy) to ensure the timely and reliable delivery of quality healthcare for all (health). The alignment of the environment–economic–health nexus enables global advancement of the integrated and interdependent SDGs. The country context therefore needs to be considered when advancing the SDGs. In low‐ and middle‐income countries (LMICs), there are often infrastructural and economic lags that limit healthcare delivery. For the rollout of UHC, some LMICs often require preliminary steps and additional resources to establish adequate infrastructure, which requires extra capital investment, before they are suitably equipped to deliver decent healthcare.

After considering the role of the environment–economic–health nexus, health (SDG 3; which includes UHC) also needs to be viewed in the context of the other SDGs to reflect its integration and interdependence, like all the other SDGs. Basic needs are to be met for effective health coverage such as alleviating poverty (SDG 1) and hunger (SDG 2), family planning (aligned to SDG 5 on gender equality), providing decent jobs and growing the economy (SDG 8), and developing adequate infrastructure (SDG 9).^3^ In addition, supporting SDGs to enable the realization of SDG 3 goals are maintaining a sustainable environment with responsible production and consumption (SDGs 11 and 12).^3^ Hence, achieving SDG 3's health targets is dependent on the other influencing SDGs that regulate implementation, i.e., advance or constrain progress. Similarly, SDG 3 also impacts the progression of the other SDGs. This reflects the integration and interdependence of the SDGs. A robustly aligned and enabling economic–environment–health nexus is thus critical for SDG implementation.

Given the integration and interdependency of the SDGs, investment solely in the health sector is therefore insufficient to achieve the global health targets^4^ of SDG 3. Investments in all related and relevant sectors that are interdependent will better improve the probability of realizing the SDGs. Cross‐sector investing will result in synergistic outcomes. For example, investment in improving public transportation with easy access and extensive networks and open, safe recreational areas (i.e., providing an enabling environment) will ripple to improve physical activity with less reliance on private transport. Further, improving the education of citizens (SDG 4) by equipping them with relevant and needed skills helps to reduce poverty (SDG 1) by improving employment rates (SDG 8), thereby increasing the economically active population, and ultimately sustaining the gross domestic product (GDP). Quality education is thus critical to sustain economic growth. Therefore, SDG integration and interdependence are fundamental, and SDGs should not be viewed in isolation. The integration and interdependencies of the SDGs, through synergism leveraged through the SDG ecosystem, drive advancement.

The SDGs are global and complex with varying degrees of success in their implementation. The countries that implement the SDGs vary in their ability to make steady progress and deliver. Therefore, an agile SDG implementation framework is needed that can be adapted according to each country's ability for advancing the SDGs.

### 1.3 Sustainable Development Goal 3: Good Health and Universal Coverage (3.8)

Good health at low cost for all captures the essence of SDG 3. One of the major levers for achieving SDG 3's targets is through the implementation of SDG 3.8 on UHC, which emphasizes the right of all citizens to access quality health services without risking financial hardship.^5^ As a major global, political, and ethical health goal, UHC embraces fairness, equity, and benefit with national and international reach.^6^ Although UHC only represents one SDG 3 target, it supports and enables the advancement of the other SDG 3 targets.^7^

Global health transitions include public health improvements (e.g., basic sewage and sanitation), the epidemiological transition (that reduced the burden of communicable diseases),^8^ and potentially UHC. Universal access is defined as no barriers to care, such as geographic, financial, organizational, sociocultural, and gender‐based constraints.^9^ Health expenses should be charged according to socioeconomic status to ensure that poor households are not disproportionately burdened relative to wealthier households.^10^ Therefore, basic health services (at least) should be free for poor citizens with less indigent citizens heavily subsidized.^11^ Health services also encompass curative care (for individuals) and health promotion (for communities)^12^ which alleviate the burden on healthcare service delivery. Thus, the return on investment in health delivers healthier citizens with increased life expectancy, economic activity, and productivity^13^ (due to healthier economically active populations) with less burden on health service delivery due to fewer patients. A healthy population therefore better serves economic development provided that an enabling environment exists, and the correct skill set is available to meet the employment demands in a particular country. This reflects the necessity for a robustly aligned environment–economic–health nexus to support SDG integration and interdependence during implementation.

### 1.4 People‐Centric Health Services Delivery through Universal Health Coverage Implementation

Within the context of UHC, it is imperative to understand the global strategy of health service delivery. The WHO global strategy on integrated people‐centered health services lays out the fundamentals of progressive health services for modern needs. Key principles are to embed a person‐centered vision, which relates to the patient experience and outcomes, healthcare access to all, given that ≈1 billion people have no access to adequate healthcare, a focus on the life course (from fetal life to ageing), and effective, efficient, safe, and timely healthcare delivery.^14^ The key deliverable from the WHO global strategy on integrated people‐centered health services is the implementation of UHC. UHC aims for all people to have access to healthcare without the risk of financial distress.^14^ Health promotion and prevention is the focus above treatment.

For UHC implementation, service delivery improvements are continuous. There is a shift from fragmentation to coordinated, collaborative partnerships. Each country is responsible for rolling out UHC, often financed through national health insurance schemes. The introduction of national health insurance schemes should be phased in for resilience during economic downturns, and only scaled up during expansionary phases.^15^ The implementation of national health insurance to achieve UHC presents a major challenge in resource‐constrained LMICs. However, there are successes such as Rwanda where UHC has been implemented with great coverage. Rwanda mainly provides UHC to poor citizens in the informal sector through community‐based health insurance with the highest enrolment in sub‐Saharan Africa (SSA) of ≈87%.^16^ Other SSA countries lag in health insurance enrolment and have varying success: Gabon with 45%, Ghana with 38%, Senegal with 32%, Burundi with 25%, Namibia with 18%, Botswana and Kenya each with 17%, South Africa with 16%, Tanzania with 10%, Ethiopia with ≈8%, Nigeria with 3%, and Lesotho with 2%.^16^

## 2 Sustainable Development Goal Implementation

### 2.1 Framework

**Figure 1:**
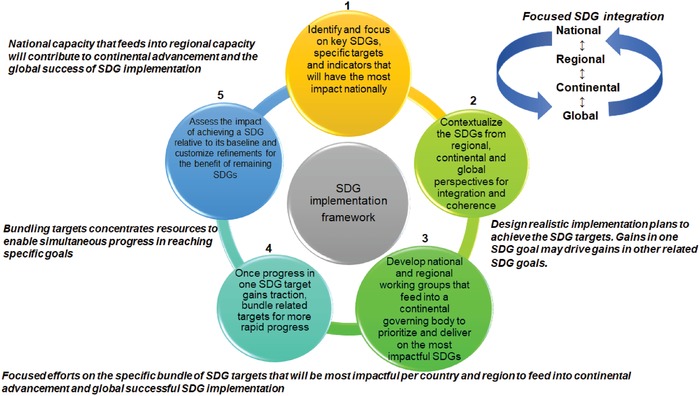
Sustainable development goal implementation framework. Sustainable development goals (SDGs) can be implemented in five distinct phases viz. i) identifying (and focusing), and ii) contextualizing the SDGs, iii) developing SDG working groups, iv) bundling the SDGs, and v) assessing their impact. This general implementation framework can be applied to SDGs and targets, e.g., SDG 3 on health and its target viz. universal health coverage.

An SDG implementation framework with five distinct phases is outlined in **Figure** **1** that each focus on (i) SDG 3 in the context of the other SDGs and (ii) the implementation of UHC. The foundational elements for the SDG framework are the robust alignment of the environment–economic–health nexus that provides adequate infrastructure (health centers that are suitably equipped with information and communications technology (ICT) and efficient networks to enable great coverage) (environment), sufficient funding to sustain healthcare delivery with limited donor reliance (economic), and well‐skilled healthcare workers with requisite skills to meet the national health demands (health).

The working, agile SDG implementation framework presented is relevant as it can be applied across all the SDGs to advance the targets and is independent of socioeconomic status; therefore, LMICs can benefit from its implementation. In health and UHC advancement, this SDG implementation framework can be adapted and used for planning and be revised and refined over the SDG era as new knowledge is generated and there are advancements in meeting the targets at national, regional, continental, and global levels.

The implementation of the SDGs presents major and complex challenges at (sub)national, regional, continental, and global levels but will reap rewards, even without full implementation and reaching of the targets. The SDGs will be met through coordinated alignment and delivery at (sub)national, regional, and continental levels.

To initiate the cascade, there needs to be a clear identification of the SDGs that will have the greatest national impact. For sustainable global health, governments should allocate and utilize their domestic resources optimally, and consider citizens' interests and open access to information, education,^13^ and awareness. For implementing UHC, the diseases with the greatest burden on citizens should be targeted and prioritized to reduce morbidity and mortality. The resources, i.e., human, financial, and infrastructural capital, should be adequate to enable advancement.

The second phase is focused SDG integration, i.e., prioritizing the key goals for implementation interdependent of related goals for increased leverage and impact. Identifying and focusing on the key SDGs at a national level, with assigned specific targets and indicators that will have the most impact, are critical. National decision making should be informed by fairness, accountability, good governance, transparency,^17^ and equity. This requires the designing of realistic implementation plans to achieve the SDGs. The domestication of the SDGs at a national level is important as this determines the priority of the goals and realistically aligns them to regional, continental, and global health goals. These nationally prioritized SDGs should then be contextualized from regional, continental, and global perspectives for enhanced integration and coherence. For UHC implementation, a realistic phase‐in approach is required where high patient populations and/or districts that are underresourced should be prioritized. Also, the geographic context should be considered when implementing UHC, given the resourcing disparities in urban and rural communities. Nationally, burdens of disease vary per province (state), but common diseases will consistently prevail. The national disease thread, i.e., the basket of high burden common diseases across the provinces, should be addressed for the most impact. Responsive, robust, and resilient health systems and resourcing will enable UHC.

The third phase is to develop national and regional working groups that feed into a continental working group (that serves a convening and coordinating function) to prioritize and deliver on the most impactful SDGs. For example, in Africa, Southern African Development Community (SADC) countries, eastern African countries, west African countries, north African countries, and SSA countries (which constitute the SADC countries and most African countries) all represent diverse nations with overlapping and distinctive SDG challenges. Therefore, strong representation from these African regions can inform continental SDG advancement. For implementing UHC, the national successes should be standardized regionally and minimally adapted per nation to ensure reproducibility. In LMICs, great gains in UHC implementation will ultimately improve citizens' quality of care. Healthier citizens may be more productive and economically active. Further, with successful UHC implementation, infrastructure is maintained, enhanced, or even built, and with the sharing of knowledge, transferring of skills, and task shifting, health practitioners are upskilled and gain relevant experience. These resourcing levers enable and reinforce UHC implementation.

In the fourth phase, gains in one SDG may drive gains in related SDGs, reflecting the influencing and interdependent nature of the integrated SDGs. Once progress in one SDG target gains traction, bundling of related targets will result in more rapid progress. The bundling of SDG targets concentrates resources to enable simultaneous progress in reaching specific goals for efficient implementation. Some SDGs influence UHC implementation. For example, if poverty (SDG 1) is pervasive it will limit UHC implementation, as poorer nations are likely financially constrained and may lack basic infrastructure. Similarly, few or poorly trained health practitioners (SDG 4) will not be able to deliver effective quality healthcare, thereby constraining progress.

The final phase of SDG implementation assesses the impact of achieving a specific SDG relative to its baseline indicators and customizing refinements will facilitate implementation of the remaining SDGs. The continuous monitoring and improvement of health service delivery efficiencies are necessary. Further, the early adoption of enabling health services support and technology to deliver integrated healthcare is essential. Robust clinical and corporate governance is necessary. For health system strengthening, resilience with distributed leadership will improve delivery and health outcomes. The implementation of UHC requires strong drive and accountability to ensure a steady progress.

National capacity that feeds into regional capacity will contribute to continental advancement and global success of SDG implementation. Focused efforts on specific bundles of SDG targets that will be most impactful per country, region, and continent to feed into global successful SDG implementation are critical. To achieve UHC, a global effort is required where timely information is shared and nations partner to achieve this important global health target. In LMICs especially, the learning and sharing of experiences and knowledge are essential throughout the course of implementation to not leave anyone behind.

### 2.2 Enabling Factors for Sustainable Development Goal Implementation

For achieving SDG 3 targets, including UHC, there are several enabling success factors. These include effective coordination, championship, and stewardship with decisive priority setting. Adequate and relevant training, skills transfer, and mentoring are crucial to grow critical mass and skills to respond to domestic health needs. UHC encompasses creating an enabling health services environment to improve patient experience and outcome; enhancing health services through robust clinical and corporate governance; improved coordination; growing critical mass; applying enabling processes to realize effective healthcare and cost efficiencies; developing, maintaining, and retaining healthcare workers; establishing resilient health systems that respond to the dynamic demands for providing excellent service; and recognizing health as part of a system that is interdependent and reciprocally influenced by other parts of that synergistic SDG ecosystem.

Other enabling factors for implementing UHC include accountability, multisector engagement, and the alignment of stakeholder action.^18^ Although the global health community increasingly endorses the sector‐wide planning for health system strengthening for realizing UHC, the enrolment of policy makers still lags.^19^ Better coordination and governance between related sectors are critical for overcoming the social, economic, environmental and political determinants of health.^20^ Public–public, public–private, and private–private health sector investments in support of UHC will improve economic growth (SDG 8).^13^ Collaborative partnerships (SDG 17) need to be fostered, including public–private, public–public, and private–private ventures. Robust and resilient public–public, public–private, and private–private partnerships will build on a shared vision to achieve the SDGs. This is particularly important to avoid donor dependence in LMICs and prepare for sustaining health systems when donors exit to pursue other ventures.

For effective implementation and realization of SDG 3 and UHC, health financing through public–public, public–private, and private–private sources should be simultaneously pursued. As SDGs are integrated and interdependent, through public–public initiatives, governmental health and aligned departments (such as economic and financial affairs (treasury, trade, and industry), social development (public service and administration), commerce, education, labor, communication, networks, and technology) should all contribute by providing a sustainable environmental (infrastructure), economic, and health platform for SDG implementation. For public–private initiatives, companies should coinvest and collaborate with public departments by investing in health or relevant infrastructure through incentivized schemes and accessible rebates. For private–private initiatives, companies can partner to enable health service delivery such as the utilization of enabling ICT infrastructure to generate reliable, timely, and robust health data for allocating health resources and revealing health trends.

### 2.3 Further Considerations for Universal Health Coverage Implementation

It is critical to reflect on some factors that may constrain UHC implementation. The rate of UHC (and SDG) implementation progress is determined by how well a country is geared for delivery. Well‐resourced national, provincial, and subprovincial (district) health departments (and sub or specialty departments) are the soft infrastructure required to primarily deliver UHC and need proper buildings, ICT, and transport networks (hard infrastructure) to provide an enabling environment for delivery. Adequate and frequent investment is required to ensure that the hard infrastructure is available and updated to enable the soft infrastructure to function optimally. This reflects the importance of robust alignment of the environment–economic–health nexus to support implementation of the integrated and interdependent SDGs.

For health systems to deliver effectively, a facilitative environment is required to enable function, and adequate economic investment is necessary to provide this supportive environment for quality healthcare delivery. In LMICs, sustainability post donor exit presents a threat for maintaining enabling infrastructure and adequate investment to continue quality healthcare delivery. Ideally, capacity should be developed to ensure that prioritized healthcare delivery needs are met. A misalignment in the environment–economic–health nexus presents a major challenge for LMICs to overcome when implementing UHC, SDG 3, and other SDGs. A misaligned environment–economic–health nexus will disrupt SDG integration and interdependence that will constrain progress.

There are several healthcare investment challenges for governments to manage. National health funding is often informed by the prevailing disease burden, stakeholder coordination is fragmented, and there may be misalignment with national health strategies and plans.^13^ Also, LMICs may not have invested adequately and more long term to sustain current and future health systems.^13^ Further, there are human capital resourcing inflexibilities such as healthcare role production and allocation, and potentially managements' vested interests in health services.^13^ The SDGs present the opportunity to address these challenges and garner political commitment in support of a mutually shared health system strengthening agenda.^13^ To deliver on the UHC goals, guidance and promotion on a coherent and consolidated agenda for health system strengthening are key for implementation through country‐specific UHC roadmaps.^13^

## 3 Conclusion

An agile SDG framework has been introduced for broad application for the implementation of the integrated and interdependent SDGs, which focuses on SDG 3 as a goal and UHC as a target. The robust alignment of the environment–economic–health nexus of the integrated and interdependent SDGs is required to enable implementation. This SDG implementation framework is particularly relevant and useful in LMIC settings that are resource constrained to guide advancement.

Good health and universal coverage for citizens captures the essence of SDG 3 and is based on a people‐centric global health services strategy. Despite health system inefficiencies, adequate healthcare infrastructure, investment, and resourcing will help to improve patients' experiences and outcomes. Focused efforts on specific bundles of SDG targets that will be most impactful per country and region will feed into continental development, thereby contributing to successful global SDG implementation. This will have a marked impact on advancing progress in LMICs. No person or country should be left behind.

## Conflict of Interest

The authors declare no conflict of interest.
